# Use of direct oral anticoagulants in primary care: a qualitative study integrating patient and practitioner perspectives

**DOI:** 10.3399/BJGPO.2021.0226

**Published:** 2022-09-21

**Authors:** Yeyenta Mina Osasu, Caroline Mitchell, Richard Cooper

**Affiliations:** 1 Academic Unit of Primary Medical Care Faculty of Medicine, Dentistry and Health University of Sheffield, Sheffield, UK; 2 School of Health and Related Research (ScHARR), University of Sheffield, Sheffield, UK

**Keywords:** atrial fibrillation, clinicians, pharmacists, primary health care, anticoagulants, general practice

## Abstract

**Background:**

Older patients with atrial fibrillation (AF) are increasingly offered direct oral anticoagulants (DOACs) to reduce the risk of catastrophic stroke, but clinical follow-up and compliance checks are still required to maintain patient safety. Although a recent qualitative meta-analysis has explored up-to-date research in this area, little is known qualitatively about clinicians’ or patients’ views and experiences of DOAC use in primary care in the UK.

**Aim:**

To understand the experiences of healthcare practitioners and patients in relation to DOAC use in UK primary care.

**Design & setting:**

Semi-structured interviews were undertaken. Sixteen older patients with AF taking DOACs, 10 pharmacists, and six GPs were interviewed in Sheffield, England in 2018.

**Method:**

Interview questions were developed following a systematic literature review. Interviews were audio-recorded, transcribed, and analysed using six-stage thematic analysis.

**Results:**

The integrated perspectives show that all three participant groups preferred DOACs over warfarin, a preference driven mainly by the safety profile compared with warfarin. GPs valued pharmacists' input in anticoagulant care, and pharmacists discussed patient safety in the context of anticoagulant audits, and highlighted the need for continuous patient education and counselling. Medication reviews by pharmacists were seen as a positive contribution to medicines optimisation.

**Conclusion:**

Patients had an overriding trust in their doctors. GPs valued a collaborative approach with other clinicians, and community pharmacists appeared to highlight operational challenges in primary care that may limit the effectiveness of interventions.

## How this fits in

Clinicians’ experiences regarding oral anticoagulant prescribing for patients with AF have been explored previously.^1^ Uncertainty and bleeding risks are still associated with DOACs despite their efficacy and safety profile.^2^ Reduced monitoring results in fewer encounters for opportunistic review and reinforcement of safety messages, especially in older patients with comorbidities.^2^ This research integrates the perspectives of clinicians, pharmacists, and patients in anticoagulant optimisation in primary care.

## Introduction

Increasingly, older patients with AF are being prescribed DOACs to reduce the risk of catastrophic stroke.^3^ Although a recent qualitative meta-analysis has explored up-to-date research in this area,^1^ there is sparse knowledge in the UK about clinicians’ or patients’ views and experiences of DOAC use in primary care.

The authors recently reviewed international literature on patient and professional perspectives on the safety and effectiveness of anticoagulants, and identified a gap in the literature.^4^ The aim of this research is to understand the experiences of healthcare practitioners and patients in relation to DOAC use in primary care.

## Method

A qualitative study was undertaken, using semi-structured interviews. The researcher is a clinical pharmacist with previous experience in anticoagulation and working with the older people. Semi-structured interviews were conducted with GPs, pharmacists, and patients.

### Setting

Interviews were conducted in patients’ homes, GP surgeries, and community pharmacies across Sheffield, UK. Sheffield’s marked difference in socioeconomic status and ethnic variation made the city an ideal location for purposive and maximum variation sampling.

### Sampling

Twenty-five GP practices were invited to participate via National Institute for Health and Care Research (NIHR) Clinical Research Network (CRN) Yorkshire and Humber. Participating GP practices were purposively sampled according to their size and indices of multiple deprivation profiles.^5^ The prescribing lead GP and community pharmacist in closest proximity to participating surgeries were interviewed.

Older patients aged ≥65 years and diagnosed with AF, with at least one comorbidity and registered in the participating GP surgery in Sheffield, were invited. Patients who wished to participate responded by post and were chosen randomly from each age bracket.

The sample size was not determined in advance, but all participants were interviewed, and analysis continued using a constant comparative approach until no new themes were identified.^6^ This was determined when additional data no longer provided new insights to the analysis. All through the process, the demographic make-up of the sample was regularly reviewed by checking variation by sex and age as stipulated in the recruitment protocol.^7^

### Data collection and processing

Semi-structured interviews were conducted based on the topic guide, which was developed after reviewing the literature and after feedback from a public interest group (Patient and Public Involvement [PPI]). All interviews were conducted by YO and consent was sought before starting each interview. Patient interviews lasted 45–60 minutes. GP and pharmacist interviews lasted about 30–45 minutes.

Interviews were recorded using an encrypted digital audio-recorder. Participants were anonymised and pseudonyms were used during transcription. All electronic information, including audio-recordings and participant demographic details, was stored electronically on a site file, which was stored on a password-protected and encrypted university computer.^8^ Recruitment and interviews were conducted from May to December 2018.

### Data analysis

Data analysis was carried out by YO using a thematic approach.^9^ This was done concurrently with data collection to enable recruitment of more participants until saturation was achieved.^10–12^

The interview topic guide was refined based on responses from previous interviews. Recorded interviews were transcribed verbatim and reread against the original audio-recordings to check for transcript accuracy. All transcripts were imported into NVivo (version 12 for Mac). There was independent verification of the emergent thematic framework, including independent analysis of transcribed data by two members of the team (CM and RC). Following analysis, the findings were also presented to members of the PPI group as feedback. The emergent themes were subjected to critical interpretative challenges during regular research meetings with CM and RC.

## Results

Thirty-two participants were recruited to the study, comprising 16 patients aged 67–89 years (Table 1), 10 pharmacists, and six GPs (Table 2). Six GP surgeries participated, comprising teaching and non-teaching practices with varying DOAC prescribing rates. Four practices and one patient withdrew after initially expressing interest. The GPs and pharmacists were heterogeneous in sex, age, and experience.

**Table 1.** Demographic information of patient participants

Patient ID code	Sex	Age, years	Comorbidities, *n*	Hospital or community initiation	Anticoagulant	Previous anticoagulant or antiplatelet	CHA_2_DS_2_VASc*	HAS- BLED^†^	IMD quintile group	IMD classification	ONSoccupation coding
PT1	F	79	10	Hospital	Apixaban	Clopidogrel	6	Not recorded	3	Average deprivation	4
PT2	M	72	3	GP	Rivaroxaban	Aspirin	1	2	5	Most deprived	4
PT3	M	81	3	GP	Apixaban	Aspirin	4	2	3	Average deprivation	3
PT4	M	89	2	Hospital	Dabigatran	Warfarin	3	2	3	Average deprivation	5
PT5	M	81	3	GP	Apixaban	Aspirin	5	1	3	Average deprivation	1
PT6	F	77	3	GP	Apixaban	None	3	Not recorded	3	Average deprivation	4
PT7	F	71	9	Hospital	Rivaroxaban	Warfarin	Not recorded	Not recorded	1	Least deprived	7
PT8	F	76	7	Hospital	Apixaban	Aspirin	4	Not recorded	2	Below average deprivation	6
PT9	M	78	3	Hospital	Apixaban	None	3	Not recorded	4	Above average deprivation	2
PT10	M	85	7	Hospital	Apixaban	Warfarin	4	3	2	Below average deprivation	1
PT11	F	80	3	GP	Apixaban	Aspirin, warfarin	Not recorded	Not recorded	5	Most deprived	9
PT12	M	67	6	GP	Apixaban	Aspirin	Not recorded	Not recorded	5	Most deprived	3
PT13	F		7	GP	Apixaban	Aspirin	2	1	1	Least deprived	9/7
PT14	M	76	4	Hospital	Apixaban	Aspirin	Not recorded	Not recorded	1	Least deprived	4
PT15	F	73	4	GP	Apixaban	None	3	1	1	Least deprived	4
PT16	F	77	6	Hospital	Apixaban	None	Not recorded	Not recorded	5	Most deprived	2

IMD = Index of Multiple Deprivation. ONS = Office for National Statistics.

*CHA2DS2VASC score = Congestive heart failure (1), Hypertension (1), Age ≥75yrs (2), Diabetes (1), Stroke or transient ischaemic attack (2), Vascular disease (1), Age ≤65yrs (1), Sex category (1).

^†^HASBLED Score = Hypertension (1), Abnormal renal/ liver function (1), Stroke (1), Bleeding history or predisposition (1), Labile INR (1), Elderly (1), Drugs/alcohol concomitantly (1).

**Table 2.** Demographics of healthcare professionals

Professional ID code	Sex	Age band, years	Year qualified	Status
**GPs**				
GP1	F	40–44	2006	Partner
GP2	M	35–39	2011	Partner
GP3	F	35–39	2011	Salaried
GP4	M	35–39	2009	Partner
GP5	M	55–59	1986	Partner
GP6	F	40–44	2006	Partner
**Pharmacists**				
Pharm1	F	30–34	2009	CCG practice pharmacist
Pharm2	F	40–44	1996	Practice employed
Pharm3	F	25–29	2012	Community
Pharm4	F	40–44	1998	Practice employed
Pharm5	M	30–34	2010	CCG practice pharmacist
Pharm6	M	35–39	2009	Community
Pharm7	M	45–49	1994	Community
Pharm8	M	40–44	1998	Community and practice
Pharm9	M	25–29	2016	Community
Pharm10	F	40–44	2006	Practice employed

CCG = clinical commissioning group.

### Integrated themes

**Figure 1.**
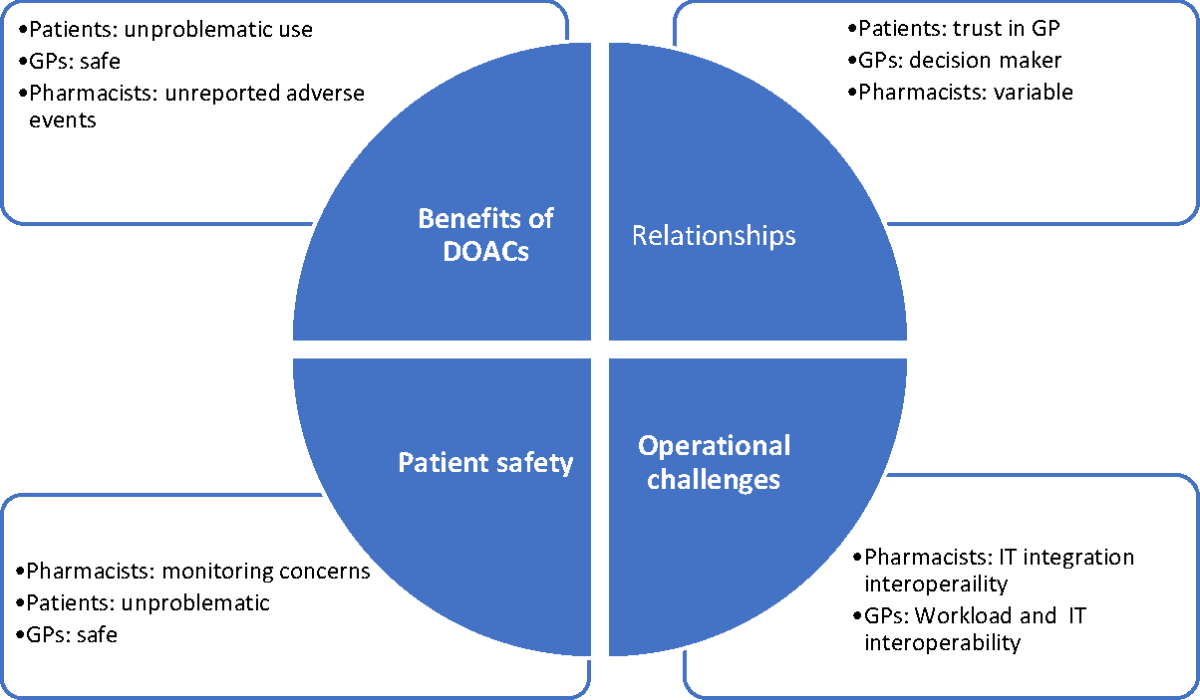
The four integrated themes and how these link to the participant groups. DOAC = direct oral anticoagulant. IT = information technology.

The integrated themes were as follows: (i) Benefits of DOACs; (ii) Patient safety; (iii) Relationships; and (iv) Operational challenges in primary care. Figure 1 is a diagrammatic representation of the integrated themes from all three participant groups.

#### Benefits of DOACs

The integrated perspectives show that all three participant groups preferred DOACs over warfarin, a preference driven mainly by the safety profile compared with warfarin. For patients, preference was driven by the tolerability of DOAC medication and the minimal impact on lifestyle, medication regimen, and management:

*'I’m quite happy with the treatment I’ve got and when you’re satisfied you don’t want to dig beneath it, it’s when you’re not satisfied that you want to dig beneath it.*' (PT9)

GPs and pharmacists reported having negligible patient complaints, and the convenience of prescribing with less complexity in anticoagulation management appeared to influence GP preference for DOACs:

*'Generally, I would much prefer* [patients] *to start on a DOAC than I would do on warfarin. Just because of the convenience*.' (GP2)

#### Patient safety

Pharmacists discussed patient safety considerations in the context of anticoagulant audits that had been carried out by practice and clinical commissioning group (CCG) pharmacists in GP practices across the city:

*' … the first bit was done 2 years ago, and then we sort of did a mop up of it just to check, but when you compare the first year to the second year, a lot less patients were on doses that needed amending. You know, it was appropriate. So it sort of showed really that prescribing has improved.'* (Pharm5, CCG pharmacist)

While auditing was a good way of monitoring prescribing on GP systems, one pharmacist described a patient-centred approach that involved speaking to the patient directly to improve health literacy, awareness, and reinforce safety messaging:

'*I think there is a concern because obviously one advantage of seeing the patient regularly is that it’s a chance to reinforce all of the health education bit, and making sure that the patient is taking the medication.'* (Pharm7, practice pharmacist)

#### Relationships

The nature of relationships between patients, GPs, and pharmacists was important in realising optimal anticoagulant therapy with DOACs. Patients’ trust in the doctor meant that they were willing to accept the GP's recommendations even when they were sceptical about the benefits of DOACs. For example, one patient put it this way:

'… *although I might say “well I’m not too keen on it”, if they say “but you will benefit from it”, then I’ll take their word for it.'* (PT12)

For other patients, this trust in the doctor appeared to result in a passive dependence on the doctor’s knowledge, which emerged in patients’ claims that they did not read patient information leaflets because they believed *'the doctor knows best’* and isn’t trying to harm them:

*‘My doctor is very easy to talk to and we did discuss it* [medication]*, but then I suppose, like all patients, you always think the doctor knows best.’* (PT 5)

GPs also acknowledged the importance of maintaining good relationships and careful consideration when discussing medication choice with patients. Some GPs adopted a decision-making role for the patients because they presumed that the information being provided was too complex for patients to understand:

*'And how are they supposed to make that decision? … okay you can give them a leaflet but a lot of them haven't got the kind of academic background, that’s really difficult stuff to ask people to do so of course they need us to guide them. So I don’t feel bad about actually giving people a push in the direction that I think is right.'* (GP3)

Meanwhile, some community pharmacists did not always feel they could access the GP to resolve queries. However, community pharmacists in close proximity to the GP (for example, adjoined to the surgery) or those who had practice pharmacists with whom they could liaise with within the GP surgery reported better working relationships.

One community pharmacist described challenges in the way they were perceived by patients and suggested that patients were often reluctant to engage with community pharmacy services, such as Medicines Use Review and New Medicine Service, because these were seen as duplicated tasks that they received from the GP:

*'I think the limitation is that normally when someone has been started on a new medication, they’re getting a lot of input from their doctor anyway, ‘cause the doctor maybe says, “Try that and come back in a few weeks and we’ll review it,” so there is a lot going on in terms of monitoring and they’re often very reluctant to speak to me.*' (Pharm9, community pharmacist)

In addition, patients preferred continued relationships with GPs owing to the high regard and trust they had for GPs compared with pharmacists:

*'I know that pharmacists are now supposed to fulfil a more extensive role, on a consultative basis, but I don’t really consult him about things at all and they’re so damn busy that they don’t talk to me about tablets. They hand over a prescription, and give me tablets, and that’s it …'* (PT10)

#### Operational challenges in primary care

GPs and pharmacists described how operational challenges in the workplace limited integration and communication between healthcare professionals with a potential impact on optimal management. This was mainly discussed in relation to workload, time pressures, and interoperability between computer systems:

*'As a general pharmacist though, you get access to the summary care records. It doesn’t always give you the information that you want, because if you have got an issue, you quite often want to actually look through to see why the doctor's done it, and you can’t get any of that from summary care.'* (Pharm8, community pharmacist)

*'The hospital elected to go for Hospital SystmOne and Lorenzo, and Hospital SystmOne and Lorenzo don’t speak to each other, and Hospital SystmOne doesn’t speak to EMIS, and Hospital SystmOne doesn’t speak very nicely to the GP SystmOne.'* (GP5)

GPs also expressed challenges relating to time and work pressures:

*'The working day, it’s so intense and you’ve got to be so efficient and so kind of lean with what you cover. So I wouldn’t bring any complications into the conversation that weren’t brought by the patient.'* (GP3)

## Discussion

### Summary

**Figure 2.**
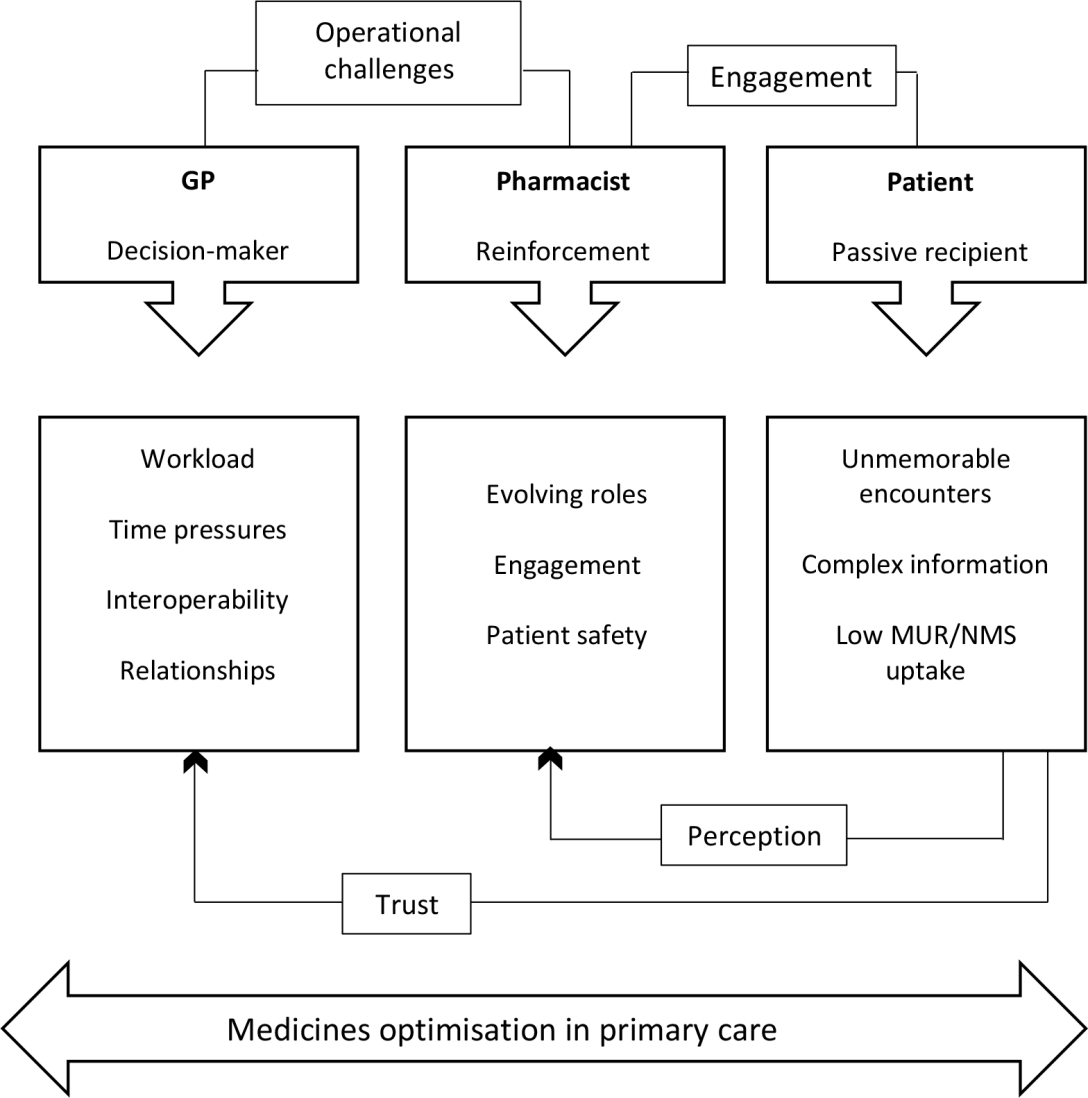
Emergent framework and the interplay between themes. NMS = New Medicine Service. MUR = Medicines Use Review.

All three participant groups highlighted the perceived benefits of DOACs. Figure 2 is a diagram showing the relationship between the dominant emerging themes. Using the example of DOACs has illustrated enduring issues in primary care, which appear to impinge on optimal medication management.

Table 1 shows that CHA_2_DS_2_-VASc and HAS-BLED scores were not always recorded in the patient’s clinical records, with possible implications to treatment optimisation and patient safety. There was no clear correlation between place of initiation and the poor recording. Although arguably beyond the scope of this research, these findings suggest areas for improvement in primary care, including interoperability, role boundaries, and organisation of work within primary care.

Hard-to-reach subpopulations may be significantly disadvantaged; for example, those with historically poorer outcomes may be digitally excluded, or unable to respond to letter, emails, or text alerts to attend medication reviews owing to poor literacy, and complex health and social needs. Medicines optimisation for patients on high-risk drugs, such as DOACs, should involve effective communication with patients to check adherence and potential side effects. Community, primary care network (PCN), and practice pharmacists are best positioned to offer medication reviews to support appropriate anticoagulant dosing based on current weight and renal function.

Although patients relied on their GPs for decision-making, the patient’s perspective of DOACs would likely vary depending on whether they had previously been on an oral anticoagulant or had a previous stroke. Before starting a DOAC, only three patients were previously on warfarin, eight on antiplatelets, four on neither, and one previously on aspirin and warfarin. Therefore, the implications for patient outcomes may be linked to poor understanding of the risks associated with anticoagulants, such as bleeding and the stroke risk associated with poor adherence.^13,14^

This research was conducted in 2018 and there is a risk that the COVID-19 pandemic presented even fewer opportunities for medication reviews, routine monitoring, and reinforced messages post-index consultations. Nevertheless, there are new models of working in primary care as services are restored, including the offer of structured medication reviews to patients. This also presents an opportunity for community pharmacists to extend their role in patient safety and medicines optimisation through more integration with GPs and practice pharmacists, facilitated by interoperability of computer systems.

### Strengths and limitations

This is the first qualitative research in the UK that has explored the perspectives of all three participant groups — patients, GPs, and pharmacists — on the optimisation of DOACs. Patients and the public were involved from the research design stage and engagement continued throughout with findings presented to the PPI group. The sample sizes of six GPs, sixteen patients, and ten pharmacists were small and resource limitations precluded a larger sample size. Nevertheless, the perspectives of patients and healthcare professionals were similar and confirmatory.

Of the four practices that withdrew, two did so because there was no funding allocation for interpreters; this was potentially a barrier to recruiting from more ethnically diverse practices and a missed opportunity for insight and inclusive research from underserved patient populations. Notwithstanding, minority ethnic patients were invited by targeted invitations via telephone and a second wave of postal invitations, but no reply was received from this patient group.

### Comparison with existing literature

This study found that great importance was placed on patient–GP relationships and trust. The subject of trust in health care has been well documented in previous research.^15^ In this study, many patients felt doctors were the experts and would make the right decisions concerning their treatment. Patient narratives in another UK qualitative study also suggested that patients preferred to be led by the doctor when making anticoagulant treatment decisions for AF.^16^ This may have been owing to a lack of suitable educational resources and decision aids to help patients understand AF and anticoagulation at the time. However, although DOACs have been used widely since, there may still be an unmet barrier in clinical practice today with consequences for patient-centred care.^17^

When discussing patient-centred care and shared decision-making, some GPs in this study assumed that their practice was patient-centred but GP narratives described a directive and paternalistic approach to consultations and decision-making. GPs presumed patient involvement in shared decision-making but their assumed decision-maker role may risk undermining patients’ values, opinions, preferences, and potentially confidence to question the clinician’s decisions.^18^

Pharmacists in this study had concerns about patient safety and some highlighted barriers to providing the necessary patient support. The benefits of integrating pharmacists into the primary care team has been demonstrated in previous studies.^19–21^ However, blurred roles in decision-making and therapy management posed a barrier for optimised anticoagulant therapy in previous qualitative studies and this was linked to patient safety and potential harm.^22,23^

### Implications for practice

This research demonstrates that patients place a high level of trust in their GP and, despite the increasing integration of pharmacists within primary care, patients prefer continuity with their GP over other healthcare professionals, such as community pharmacists. Nevertheless, GP workloads continue to rise. As the UK emerges from the COVID-19 pandemic, community pharmacists can assume more clinical roles and long-term medication management; for example, by supporting patients who have recently started medications for long-term conditions through the new medicines service. GPs could play a role in supporting integration by encouraging patients to foster positive relationships with the community pharmacy teams who can work collaboratively with primary care colleagues to improve the quality of patient care.

In this research, patients expressed dissatisfaction with the quality and quantity of information they received from healthcare professionals. Capacity and health literacy should be taken into consideration when communicating information about high-risk medication such as DOACs. Patients highlighted a need to improve the quality of patient information through making information succinct and involving patients in the development of patient-tailored medication information.

Interoperability of computer systems across healthcare settings, such as community pharmacy and GP surgery, will facilitate integration and communication between healthcare professionals to enhance patient care and medicines optimisation.

## Data

The dataset relied on in this article is available from the corresponding author on reasonable request.
